# Formation of metallic magnetic clusters in a Kondo-lattice metal: Evidence from an optical study

**DOI:** 10.1038/srep00890

**Published:** 2012-11-27

**Authors:** N. N. Kovaleva, K. I. Kugel, A. V. Bazhenov, T. N. Fursova, W. Löser, Y. Xu, G. Behr, F. V. Kusmartsev

**Affiliations:** 1 Department of Physics, Loughborough University, LE11 3TU Loughborough, United Kingdom; 2 Institute of Physics, ASCR, 18221 Prague, Czech Republic; 3 Institute for Theoretical and Applied Electrodynamics, Russian Academy of Sciences, 125412 Moscow, Russia; 4 Institute for Solid State Physics, Russian Academy of Sciences, 142432 Chernogolovka, Russia; 5 Leibniz Institut für Festkörper- und Werkstoffforschung Dresden, D-01171 Dresden, Germany; 6 State Key Laboratory of Solidification Processing, Northwestern Polytechnical University, Xi'an 710072, P. R. China; 7 Deceased

## Abstract

Magnetic materials are usually divided into two classes: those with localised magnetic moments, and those with itinerant charge carriers. We present a comprehensive experimental (spectroscopic ellipsomerty) and theoretical study to demonstrate that these two types of magnetism do not only coexist but complement each other in the Kondo-lattice metal, Tb_2_PdSi_3_. In this material the itinerant charge carriers interact with large localised magnetic moments of Tb(4f) states, forming complex magnetic lattices at low temperatures, which we associate with self-organisation of magnetic clusters. The formation of magnetic clusters results in low-energy optical spectral weight shifts, which correspond to opening of the pseudogap in the conduction band of the itinerant charge carriers and development of the low- and high-spin intersite electronic transitions. This phenomenon, driven by self-trapping of electrons by magnetic fluctuations, could be common in correlated metals, including besides Kondo-lattice metals, Fe-based and cuprate superconductors.

The quantum mechanics' Pauli principle, also known as the exclusion principle, postulates that a single orbital may accommodate no more than two electrons and, if it is doubly occupied, the electron spins must be paired. This principal is implicated in the electronic and magnetic properties of solids as the so-called *exchange effect*. In the Stoner model of ferromagnetism of weakly-correlated itinerant electrons (the form observed in the elemental metals iron, cobalt and nickel) electron bands can spontaneously split into up and down spins. This happens if the relative gain in an exchange interaction is larger than the loss in kinetic energy^1^. On the other hand, in the Heisenberg model of localised magnetism in magnetic insulators (the form observed in compounds LaMnO_3_, KCuF_3_, V_2_O_3_, and many others) the electrons, involved in the exchange coupling, are localised by strong electronic correlations when the large on-site Coulomb repulsion *U* opens a well-defined gap for charge carriers^2^,^3^,^4^; and an adequate description of the ground state properties of these systems in terms of the superexchange spin-orbital models has been suggested^5^,^6^,^7^,^8^,^9^,^10^,^11^,^12^,^13^.

However, recently synthesised Pd-based ternary compounds of the form *R*_2_PdSi_3_, where *R* is a rare earth atom^14^, have been found to have pronounced anomalies in resistivity, magnetization and susceptibility, associated in literature with their unusual or “novel” magnetic properties^15^,^16^,^17^,^18^,^19^,^20^. These compounds descend from *R*Si_2_ compounds with the layered AlB_2_-type crystal structure, and have quasi-hexagonal symmetry [see Fig. 1 (a)]. The Tb rare-earth compound reveals the most intriguing regime of quasi-one-dimensional magnetism and anisotropic “spin-glass”-like behaviour (see Refs. [15,16,17,18] and references therein). In this compound the states at the Fermi level (*E*_F_) were found to be dominated by the highly conducting itinerant electrons of the Tb 5d^1^ orbital, whereas the Tb 4f states responsible for the large magnetic moment (of 9.68 *µ_B_*, comparable to that of the free-ion Tb^3+^ value of 9.72 *µ_B_*) were found to be localised deep below *E*_F_ near 8 eV^21^. In this paper we demonstrate that the unusual magnetic properties of Tb_2_PdSi_3_ are the result of complementary effects of itinerant and localised magnetism in the Kondo-lattice metallic state. The itinerant charge carriers interact with large localised magnetic moments of the Tb 4f states, forming regular lattices of self-organised ferromagnetic clusters below the Néel temperature *T*_N_ = 23.6 K.

To elucidate the nature of electronic instabilities in the Kondo-lattice metal Tb_2_PdSi_3_, and the resulting unusual magnetic ordering phenomena, we used a comprehensive spectroscopic ellipsometry approach, which was successfully applied in our earlier studies of electronic correlations in the magnetic insulators^10^,^11^,^22^.

So far the properties of the metallic Kondo-lattice compound Tb_2_PdSi_3_ have been discussed in terms of quasi-one-dimensional magnetism at high temperatures (near 55 K) and anisotropic “spin-glass”-like behaviour at low temperatures (< 10 K)^15^,^16^,^17^,^18^. In particular, the broad peak in magnetic susceptibility near 55 K has been attributed to the magnetic correlations arising in one-dimensional spin chains, which have been intensively studied since the seminal paper by Bonner and Fischer^23^. The peak exhibits strong anisotropy in the paramagnetic state, also suggesting some similarities with quasi-one-dimensional systems. All the results convincingly demonstrate that some kind of long-range magnetic ordering sets in below *T*_N_ = 23.6 K^15^,^16^,^17^,^18^. However, the nature of the magnetism appears to be very complex, with strong evidence of ferromagnetic correlations and a complex ferrimagnetic type order in the basal plane, and a kind of anti- and ferromagnetic correlations perpendicular to the basal plane, along the **c** axis^15^.

Neutron diffraction studies of this compound in zero magnetic field revealed both the long- and short-range order^15^,^17^,^18^. The long-range order (LRO) below *T*_N_ = 23.6 K may be described as follows. The magnetic unit cell of Tb_2_PdSi_3_ is four times as big in the basal plane, contains four magnetic Tb^3+^ ions (at the 3***f*** and 1*a* site positions of *P*6/*mmm* hexagonal symmetry), and is sixteen times as big along the **c** direction. While the Tb moments are lying within the basal plane, as illustrated by Fig. 1 (b), the most intriguing is the spin sequence of the magnetic moments associated with the individual Tb^3+^ ions along the **c** direction. The neutron diffraction study^15^ suggests that for all 3***f*** positions there is a spin-sequence of eight positive orientation of the Tb moments “+”, followed by their eight negative orientation “−”. On the other hand, the magnetic moments at 1***a*** positions generate a different spin-sequence, consisting of the alternating orientations “+ + − − − − + + − − + + + + − −”, as schematically presented in Fig. 1 (c). The low-temperature short-range correlations (SRC) are suggested to arise from frustrated magnetic moments within the LRO, and are associated with the “spin-glass”-like behaviour detected in the magnetic susceptibility measurements^15^,^16^,^17^,^18^. We argue that the complex “exotic” phases observed in this compound in an external magnetic field and the low-temperature “glassy” behavior are associated with self-organisation of magnetic clusters, which can merge into string-like droplets or stripy microdomains, may be ordered or disordered.

Our main results are as follows. After a thorough study of the optical response of the Kondo-lattice metal Tb_2_PdSi_3_ at low energies, we discovered anisotropic anomalous optical spectral weight (SW) changes across the Néel temperature *T*_N_ = 23.6 K, involving the free charge carrier (Drude) resonance and two midinfrared (MIR) optical bands at 0.2 eV and 0.6 eV. We found that a vast amount of the optical SW of the free charge carriers (of the Drude peak) is shifted to the optical band at 0.2 eV. The observed anomalous behaviour in the low-energy optical conductivity spectra below 0.2 eV can be associated with the opening of a pseudogap in the conduction band and reconstruction of the Fermi surface. These anomalies are driven by electronic correlations related to the free electron instability against self-trapping in metallic ferromagnetic clusters, rather than band structure effect. Localisation of electrons in these magnetic clusters leads to development of the high-spin (HS) and low-spin (LS) inter-site electronic transitions, which were determined by their anomalous behavior in our comprehensive temperature study around *T*_N_ at 0.2 and 0.6 eV, respectively.

Finally, we note that in the optical conductivity spectra of the Fe-based superconductors, namely BaFe_2_As_2_ and Fe-chalcogenides, optical bands at around 0.2 eV and 0.6 eV, which evolve with electron- or hole- doping have been identified^24^,^25^,^26^. In addition, the midinfrared features at 0.2 eV and 0.6 eV are well-known and, according to numerous studies, are strongly pronounced in the optical conductivity spectra of all high-*T_c_* cuprates, whereas they are absent in their dielectric phases^27^,^28^. We can argue that electronic correlations lead here to the energy lowering due to self-trapping of charge carriers by magnetic fluctuations, thus giving rise to nanoscale inhomogeneities with a local magnetic ordering. The existence of these nanoscale inhomogeneities must manifest itself in the midinfrared optical conductivity as the high-spin (at around 0.2 eV) and low-spin (at around 0.6 eV) optical excitations.

## Results

### Wide-range optical response of Tb_2_PdSi_3_

Here we report the results of a spectroscopic ellipsometry study of a wide-range anisotropic dielectric response of high-quality single crystals of Tb_2_PdSi_3_. The available data on the optical properties of *R*_2_PdSi_3_ ternary silicides and their parent compounds *R*Si_2_ is limited, to the best of our knowledge, to a single study of the room-temperature optical reflectivity in GdSi_2_ and ErSi_2_ polycrystalline films^29^. The low- and high-temperature spectra of the real and imaginary parts of the dielectric function, *ε*(*ω*) = *ε*_1_(*ω*) + i*ε*_2_(*ω*), in [100] polarisation are shown in Fig. 2. They demonstrate the main features of the **a**-axis dielectric response of Tb_2_PdSi_3_. At low energies, the dielectric function is dominated by a Drude-type response due to transitions within the conduction band, characterized by the free charge carrier scattering rate, *γ*_D_, and the plasma frequency, *ω*_p_. The zoomed-in low-energy part of the dielectric function is shown in the inset, where we also show *ε*_1_(*ω*) at intermediate temperatures between 10 and 250 K. At higher energies, broad optical bands associated with interband transitions can be identified by the resonance and antiresonance features that appear at identical energies in *ε*_2_(*ω*) and *ε*_1_(*ω*), respectively, obeying the Kramers-Kronig relations. The spectra can be well described in terms of the Drude-Lorentz model^30^, with the results of the dispersion analysis summarised in Fig. 2 and in Table S1 (See Fig. S1 and Table S1 in the Supplementary Information to this paper). The optical response in the low-energy region is composed of a Drude peak and two bands centered in the MIR region at *ω*_1_ ≈ 0.2 eV and at *ω*_2_ ≈ 0.6 eV. At higher energies, we identify at least three interband transitions centered at 1.3, 1.7 and 3.4 eV. The **c**-axis dielectric function exhibits similar features in the optical response (not shown), however, compared to the **a**-axis spectra, we notice pronounced anisotropy in the range of the interband optical transitions, which appear at 2.2, 2.7 and 5.2 eV. The LDA calculations reproduce the main features of the optical response of Tb_2_PdSi_3_ and its anisotropy reasonably well.

Fig. 3 (a) shows the related **a**- and **c**-polarised optical-conductivity spectra, *σ*_1_(*ω*) = *ωε*_2_(*ω*)/(4*π*), in which the MIR optical band at *ω*_2_ ≈ 0.6 eV and the higher-energy bands are more pronounced than in *ε*_2_(*ω*). Fig. 3 (a) also reflects changes in the optical-conductivity spectra at elevated temperatures. In the **a**-polarized spectra, the high-energy optical bands at 1.3, 1.7, and 3.4 eV broaden, while their intensities decrease. The peak position of the strongly pronounced optical band at 1.7 eV exhibits a noticeable shift, and as a result, an isosbestic point appears in the temperature-dependent optical-conductivity spectra at 1.5 eV, associated with a displacement of the optical SW to lower photon energies. In the **c**-axis spectra, the broadening of the high-energy optical bands at 2.2 and 2.7 eV explains notable changes at photon energies between 1 and 2 eV.

To get a better insight into the temperature-dependence of the **a**- and **c**-polarised optical-conductivity spectra, in Fig. 3 (b, c) we plot the difference between *σ*_1_(*ω*, *T*) and the respective low-temperature spectrum, *σ*_1_(*ω*, 10 K). It can be seen here that the major temperature effects within the high-energy optical bands, discussed above, occur between 100 and 250 K and, therefore, can be associated with lattice anharmonicity effects. However, qualitatively different changes occur in the **a**- and **c**-polarised optical-conductivity spectra at low energies. Indeed, here the Drude response expands to higher photon energies with increasing temperature due to an increase in *γ*_D_, together with simultaneous changes in the intensity and width of the MIR optical bands at 0.2 and 0.6 eV. Qualitatively different changes at low energies in the **a**- and **c**-polarized spectra indicate that the temperature changes for these competing effects are polarisation dependent.

### Anomalies induced by magnetic ordering

The anomalous behavior of the optical bands at *ω*_1_ ≈ 0.2 eV and *ω*_2_ ≈ 0.6 eV at low temperatures also becomes evident close to the magnetic phase transition, near *T*_N_ = 23.6 K. Here the difference spectra exhibit a dip, followed by a maximum around 0.6 eV. This feature has an asymmetric shape, with an extended tail at higher photon energies, where the second weaker maximum shows up around 1.2 eV. The latter exhibits a similarly anomalous behavior near *T*_N_. One can compare the rearrangement of the SW due to these low-temperature anomalies with the anharmonicity coming into effect at elevated temperatures.

Fig. 4 (a, b) shows relatively weak changes in the **a**- and **c**-polarised optical-conductivity spectra at low photon energies (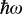 = 0.05–1.0 eV) around *T*_N_ and presents the results of the Drude-Lorentz analysis at 10 K. Contributions from the Drude term and from the MIR optical bands at 0.2 and 0.6 eV can be clearly seen here. Panels (c) and (d) of the same figure emphasise the anomalous temperature dependence of the anisotropic MIR optical conductivity near *T*_N_. The inset of Fig. 4(c) shows critical behavior of the peak intensity, 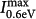, of the 0.6 eV optical band, such that 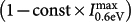 follows trends in the dc transport anomalies in the **a**-axis spectra near *T*_N_ (See Fig. S1 and Table S1 in the Supplementary Information to this paper)^31^. And finally, we analyze the difference, *σ*_1_(*ω*, 30 K) − *σ*_1_(*ω*, 10 K), of the spectra measured immediately above and well below *T*_N_, which exhibits clear anisotropy in the corresponding **a**- and **c**-polarised spectra [Fig. 4(e, f)].

According to our dispersion analysis of the **a**-axis spectra (See Fig. S1 and Table S1 in the Supplementary Information to this paper), in addition to the changes associated with the Drude response, the oscillator strength of the optical band at 0.2 eV decreases, whereas the one at 0.6 eV increases above *T*_N_. Low-energy downturn in this difference indicates that the crossing point for the SW transfer between the “head” of the Drude response and the higher-energy response is located near 0.06 eV. Above 0.06 eV, the changes due to the Drude response compete with the opposite changes associated with the MIR optical band at 0.2 eV. The cumulative changes of the Drude response and the involved MIR optical bands result in the creation of zero crossing points around 0.27 eV and 0.47 eV.

In order to quantify the SW involved in these anomalies, we have integrated the associated optical SW increment as 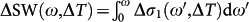. In this investigation the optical conductivity changes of the Drude response across *T*_N_, 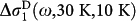, resulting from our dispersion analysis (See Fig. S1 and Table S1 in the Supplementary Information to this paper), are incorporated. For more accurate estimate of the associated energy scale, where the total optical SW obeys the f-sum rule^32^, an additional optical study at lower frequencies, which enables consistency in the dc limit *σ*_1_(*ω* → 0,*T*), is required. The resulting SW changes are presented in Fig. 4(e) in terms of the effective number of charge carriers per Tb atom, Δ*N*_eff_ = 2 *Vm*ΔSW/*πe*^2^, where *m* and *e* are the free-electron mass and charge, and 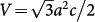 is the unit cell volume corresponding to one Tb atom, expressed through the low-temperature lattice parameters *a* = 4.0643 Å and *c* = 4.0502 Å^20^. Alternatively, we estimate the optical SW associated with the contribution of charge carriers as 
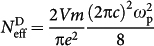
, which with *ω*_p_ = 2.703 eV gives 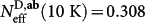 and with *ω*_p_ = 2.750 eV gives 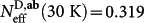 (See Fig. S1 and Table S1 in the Supplementary Information to this paper). Hence, the SW changes of the Drude response across *T*_N_, 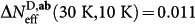, amount to 3.6% of the total low-temperature charge carrier SW, 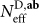 (10 K). As evident from Fig. 4 (e), these SW changes are nearly compensated around the zero-crossing point at 0.47 eV, by the opposite changes of the SW of the optical band at 0.2 eV (which are in a good quantitative agreement with the SW amplitude around 0.2 eV across the spin-density-wave (SDW) transition at *T_SDW_* = 200 K in the iron arsenide SrFe_2_As_2_^33^). Therefore, the total SW gain across *T*_N_ accumulated within the energy window 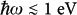 in the **ab** plane, 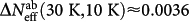 [see Fig. 4 (e)], amounts to 1.2% of the total charge carrier SW, 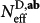 (10 K), and can be attributed to the SW gain of the optical band at 0.6 eV.

In the **c**-axis optical-conductivity spectra, in addition to the changes associated with the Drude response, the oscillator strength of the optical band at 0.2 eV decreases above *T*_N_, while the one at 0.6 eV exhibits weak temperature dependence (See Fig. S1 and Table S1 in the Supplementary Information to this paper). Our analysis represented in Fig. 4 (f) shows that the SW changes of the Drude response across *T*_N_ are nearly compensated at around 0.23 eV, by the opposite changes of the SW of the optical band at 0.2 eV. The total SW loss across *T*_N_ within the energy window 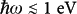 in the **c** axis amounts to 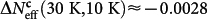 [see Fig. 4(f)].

## Discussion

**Figure 1:**
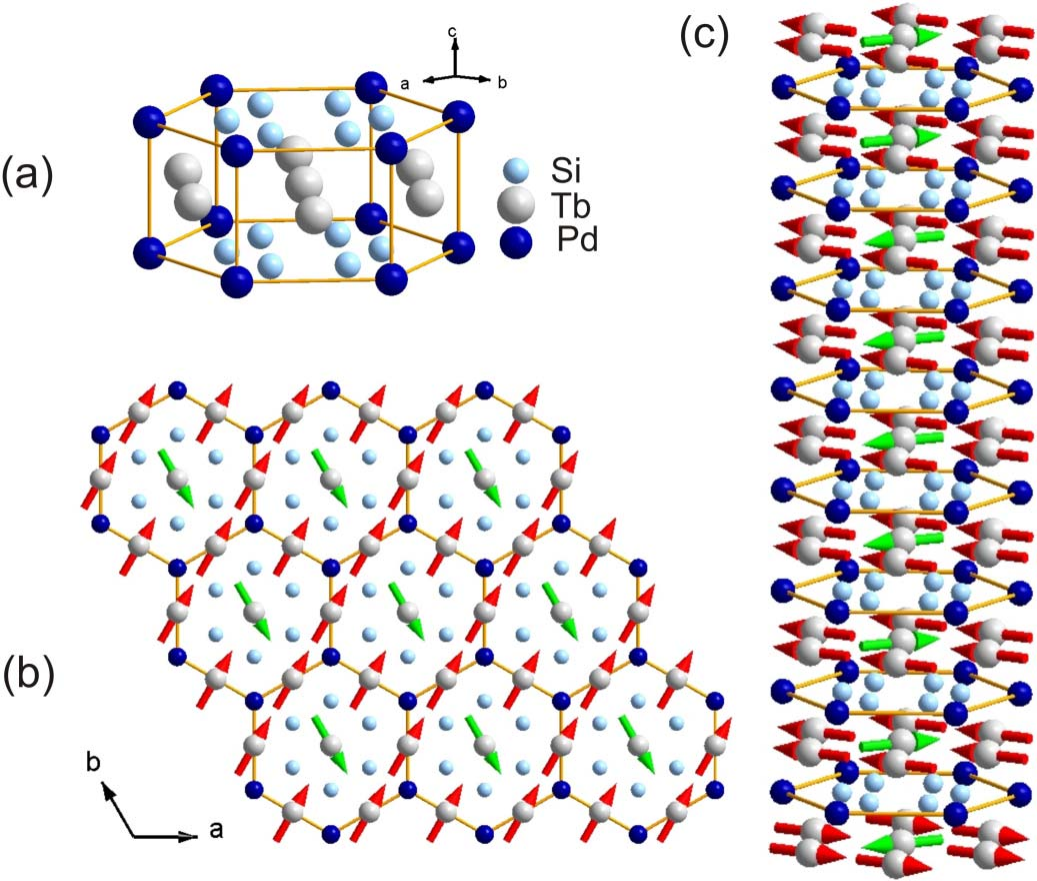
Structure and long-range magnetic order in Tb_2_PdSi_3_. (a) A simplified unit cell of Tb_2_PdSi_3_, neglecting the **c**-axis superstructure. (b,c) **ab**-plane and **c**-axis magnetic ordering patterns according to the results of neutron scattering study^15^.

**Figure 2:**
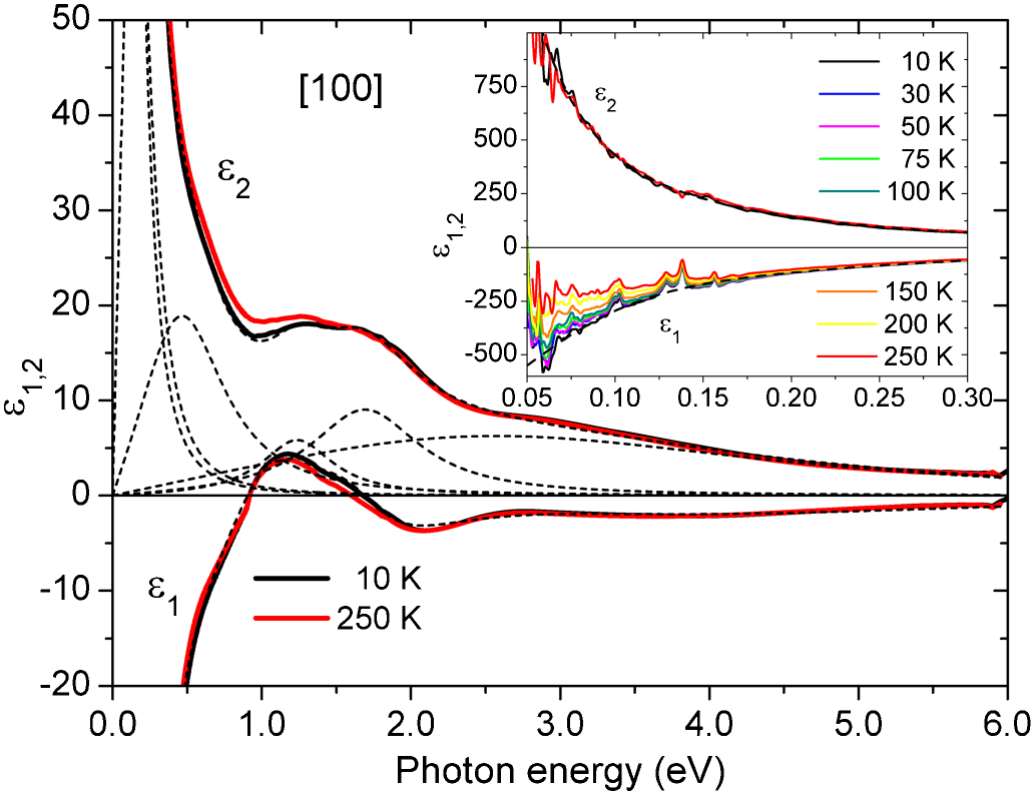
Wide-range optical response of Tb_2_PdSi_3_. Low-temperature (10 K) and high-temperature (250 K) dielectric functions of Tb_2_PdSi_3_ in [100] polarisation. Constituents of the classical dispersion analysis of the complex dielectric response at 10 K in terms of the Drude-Lorentz model^30^ are shown by dashed curves. Inset: Zoom of the low-energy Drude response.

We found that in the temperature range 10 K ≤ *T* ≤ *T*_N_, anomalous changes of the optical SW in Tb_2_PdSi_3_ occur below 1 eV, including the Drude-type resonance and the MIR optical bands at 0.2 and 0.6 eV. We attribute these anomalies to electronic instabilities (many-body effects) caused by the nature of magnetism inherent in this Kondo-lattice metal, which exhibits quasi-one-dimensional characteristics at higher temperatures and spin-glass-like behaviour at lower temperatures^15^,^16^,^17^,^18^. The complex long-range magnetic order below *T*_N_ is due to ferromagnetic correlations in the ferrimagnetic-type arrangements of the large Tb moments in the **ab** plane, and antiferromagnetic correlations between the ferromagnetic clusters, alternatively ordered along the **c** axis [see Fig. 1(b,c) ]^15^. The observed anomalous behaviour in the low-energy optical conductivity spectra below 0.2 eV can be associated with the opening of a pseudogap in the free charge carriers (Drude-type) response. As a result, the vast amount of optical SW of 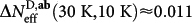 is shifted to the optical band at 0.2 eV, which we relate to electronic instability of the free charge carriers against the long-range ferromagnetic correlations in the **ab** plane below *T*_N_. The estimated amount of optical SW corresponds to a kinetic energy loss of the free charge carriers of about 5 meV, which correlates well with the characteristic temperature of the broad peak (55 K) in the **c**-axis ac susceptibility^15^,^16^,^17^,^18^. This observation can explain the quasi-one-dimensional character this Kondo-lattice metallic system displays above the Néel temperature, and suggests the existence of the chain-type ferromagnetic clusters in this temperature range.

**Figure 3:**
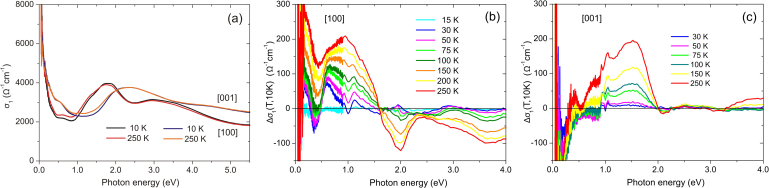
Temperature-dependent anisotropic optical conductivity. (a) Low-temperature (10 K) and high-temperature (250 K) optical-conductivity spectra, *σ*_1_(*ω*), and their anisotropy in [100] and [001] polarisations. (b, c) Details of the temperature dependence of *σ*_1_(*ω*,*T*) in the difference spectra, *σ*_1_(*ω*, *T*) − *σ*_1_(*ω*, 10 K), in [100] and [001] polarisations, respectively.

**Figure 4:**
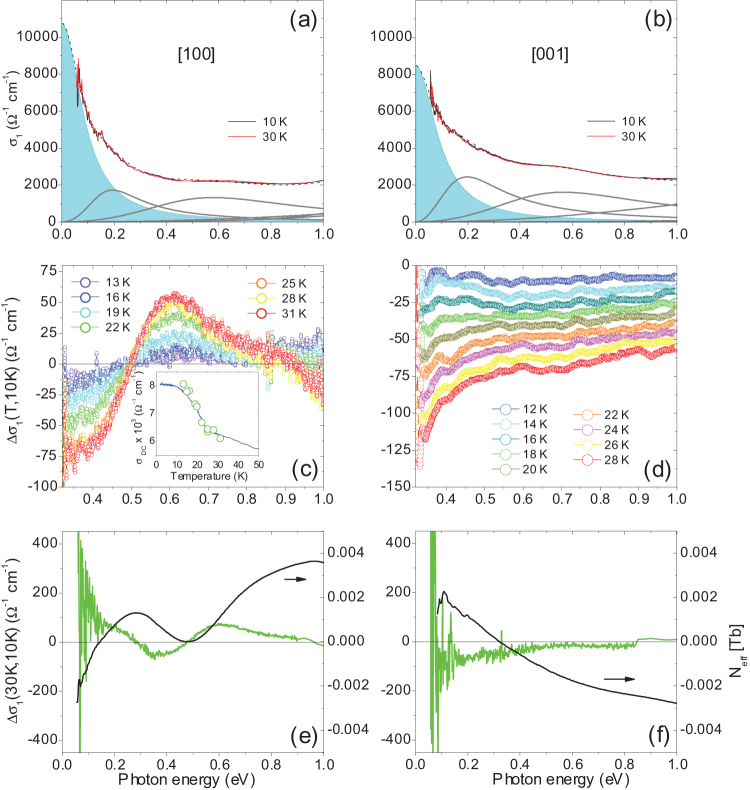
Anomalies in optical conductivity induced by magnetic ordering. Left and right panels correspond to [100] and [001] polarisations, respectively. (a, b) Low-temperature (10 and 30 K) optical-conductivity spectra, *σ*_1_(*ω*), the contribution from the Drude term (shaded area), and the Lorentz optical bands below 1 eV. (c, d) Critical behavior of the MIR optical conductivity across *T*_N_. Inset: 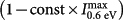 superimposed on a linear scale with the **a**-axis dc conductivity (See Fig. S1 and Table S1 in the Supplementary Information to this paper)^31^. (e, f) Δ*σ*_1_(30 K, 10 K) (green curve) and the associated SW changes (black curve).

Nevertheless, several features of the observed low-energy optical conductivity anomalies are difficult to reconcile with a simple spin-density-wave scenario, where the SW, lost below the gap, accumulates above the gap. While in the **ab** plane, the SW changes of the Drude response across *T*_N_ are nearly compensated by the opposite changes of the SW of the optical band at 0.2 eV; and the SW accumulated by the optical band at 0.6 eV, 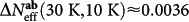 [see Fig. 4(e)], must be compensated at higher energies, which is in agreement with experimental observations in Fe-based superconductors and their parent compounds reported previously^26^,^34^. This effect, which is accompanied by the low-energy optical-conductivity anomalies, requires further experimental and theoretical study. Additionally, the anomalous increase in the dc conductivity in the **ab** plane below *T*_N_ (See Fig. S1 and Table S1 in the Supplementary Information to this paper)^31^ can be largely accounted for by the reduced scattering rate of the charge carriers due to freezing out of the spin-flip scattering, caused by ferromagnetic ordering of the Tb 4*f* moments in the formed clusters. Interestingly, as we have discovered, the intensity of the 0.6 eV band simultaneously shows an anomalous decrease below *T*_N_, indicating that the associated electronic excitation is suppressed by the frozen ferromagnetic alignment of the localised magnetic moments in the **ab** plane [see inset of Fig. 3(c)]. We attribute this behaviour to the inter-site low-spin (LS) electronic excitation in an electron pair, where two electrons with antiparallel spins are residing on the same lattice site. An opposite observable trend in the behavior of the 0.2 eV band suggests that its origin can probably be attribute to the inter-site high-spin (HS) electronic excitation. In this case, two electrons with parallel spins are residing on the same lattice site; and the associated electronic excitation is enhanced by the frozen ferromagnetic alignment of the localised magnetic moments in the **ab** plane. According to our earlier optical study of the insulating *d*^1^ (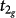) system YTiO_3_^22^, the contribution from the inter-site 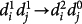 charge excitations to the optical response leads to four different excited states: a HS ^3^*T*_1_ state at energy U* − 3*J_H_*, two degenerate LS states ^1^*T*_2_ and ^1^*E* at energy U* − *J_H_*, and a LS state ^1^*A*_1_ at energy U* + 2*J_H_*^9^, where U* is the effective Coulomb repulsion of the two electrons with opposite spins on the same orbital, and *J_H_* is the Hund's rule coupling constant. There is an obvious analogy between 5d^1^ system in Tb_2_PdSi_3_ and 3d^1^ system in YTiO_3_. Following that, we assign 0.2 eV to the HS-state transition and the 0.6 eV to the first LS state, and estimate the values of U* at ≈ 0.8 eV and *J_H_* at ≈ 0.2 eV. Thus, the next LS transition is expected at ~ 1.2 eV, in a good agreement with our observations [see Fig. 3 (b)].

Having analysed the observed anomalous low-energy optical-conductivity behavior in a Kondo-lattice metal Tb_2_PdSi_3_ across *T*_N_, we discovered that the SW changes of the optical band at 0.2 eV are in a quantitative agreement with the SW amplitude around 0.2 eV across the spin-density-wave (SDW) transition at *T_SDW_* = 200 K in the iron arsenide SrFe_2_As_2_^33^. In this compound, the ground state of the antiferromagnetic SDW instability of a stripe-type (or collinear) spin configuration was suggested to result from the nesting between the hole-and-electron Fermi surfaces of itinerant electrons, rather than the superexchange interaction mediated through the off-plane As atoms^24^. The origin of the low-energy twogap behavior, found in optical measurements, and of the higher-energy 0.6 eV optical feature is still unclear^24^, which makes the nature of the driving force of the SDW instability in the iron arsenide compounds an open and a challenging question. Hence, some close qualitative and quantitative similarities with the optical features discussed in this optical study of the Kondo-lattice metal Tb_2_PdSi_3_ could be of a significant importance.

We note, however, that in the system we chose to study, the magnetic pattern is more complicated than a regular spin-density wave. Indeed, the long-range magnetic order arising at *T*_N_ is characterized by different periods along the Tb 3*f* and 1*a* chains, whereas the short-range correlations seen below *T*_N_ distort even this not particularly regular order. This is an additional evidence that we deal here with quite an irregular structure, most probably formed by spin chains of different lengths, which become frozen at lower temperatures, producing a spin-glass-like state observed in neutron diffraction experiments.

We conclude that the observed anomalous low-energy optical-conductivity behavior in a Kondo-lattice metal Tb_2_PdSi_3_ occurs due to electron correlation rather than a band structure effect. We propose that magnetism of the large localised Tb 4f magnetic moments in the metallic ferromagnetic cluster is driven by the electron exchange instability at the Fermi level, similarly to instability of itinerant electrons. The optical SW redistribution involving the free charge carrier Drude resonance (broad and narrow), and the midinfrared high-spin-(HS) and low-spin- (LS) state optical bands at 0.2 eV and 0.6 eV respectively, indicates electronic instability of the free charge carriers against coupling in the metallic ferromagnetic cluster. Thus, by observing the associated low-energy optical conductivity anomalies, one may elucidate further the evolution in the creation of magnetic clusters and estimate the amount of free carriers self-trapped by them in the Kondo-lattice metal.

In the first instance the magnetic clusters may arise from magnetic polarons^35^,^36^,^37^,^38^, which are formed by large moments and free carriers. It is well known that small polarons in general may form string-like objects^39^,^40^,^41^. In the present compound the magnetic polarons may display similar behaviour dictated by a large anisotropy and the quasi-one dimensional character of the magnetic ordering^15^,^16^,^17^,^18^. Here, the lowest energy of the localised states corresponds to the small polarons assembled into ferromagnetic string clusters. In a single cluster, many free particles are self-trapped by the spin fluctuation involving many large Tb magnetic moments having the spin orientation parallel to the **ab** plane. The spins of the trapped charge carriers and magnetic moments are collinear.

**Figure 5:**
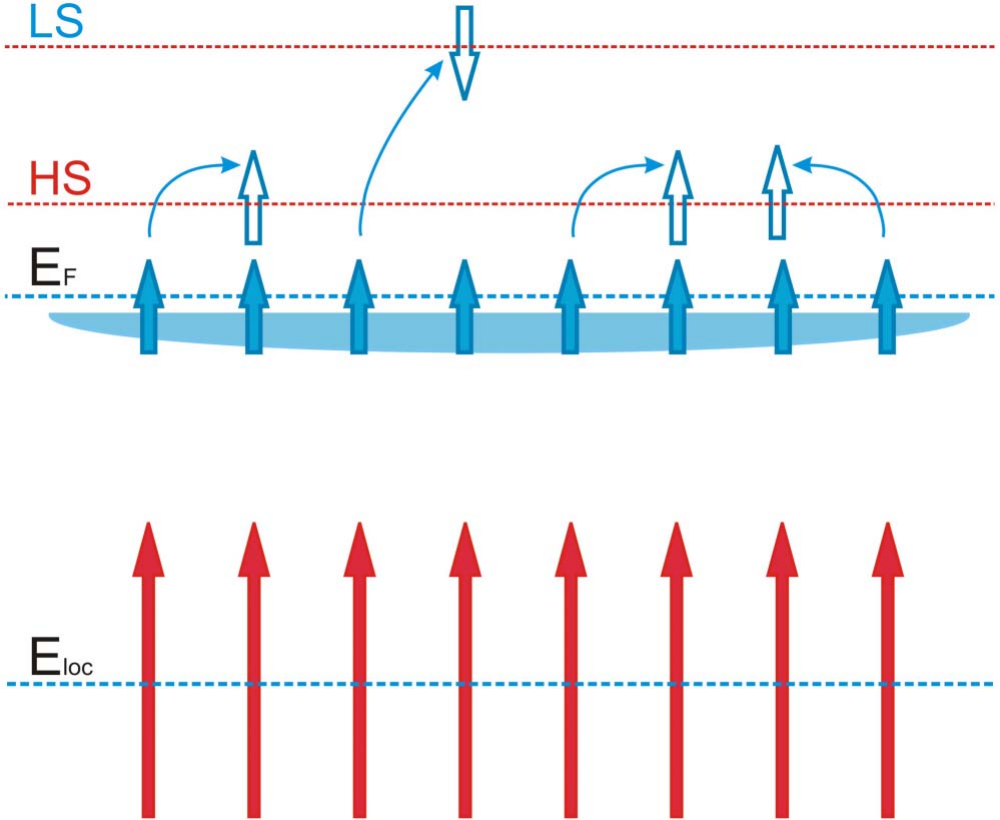
A schematic representation of ferromagnetic metallic cluster in Tb_2_PdSi_3_. Localisation of itinerant electrons in the string cluster is associated with development of the high-spin (HS) and low-spin (LS) inter-site electronic transitions.

We estimated the size of these ferromagnetic string clusters, taking into account many-body effects of Coulomb interaction between electrons, their interaction with localised large moments, which are ferromagnetically ordered and coupled to each other due to some indirect exchange interaction, induced by itinerant charge carriers. The detailed mechanism of the string clusters formation is discussed in the Supplementary Information, here we only describe its main features. According to this mechanism, the itinerant charge carriers and the localised moments are forming together a potential well, where these charge carriers are self-trapped. Due to such a localisation, the kinetic energy of the trapped electrons increases, while at the same time their potential energy decreases. As a result, the total energy of the electrons in the clusters becomes smaller or comparable with the energy of itinerant charge carriers. The comparison indicates that a string with many trapped particles and magnetic moments may have lower total energy (including electronic, magnetic, and Coulomb contributions), as shown schematically in Fig. 5.

It is important to note that the energies of string clusters having different lengths are very close to each other. In general, we expect to find a very broad distribution of such clusters at the temperature above *T*_N_ and, possibly, also below this temperature. Such magnetic clusters give rise to the tail in the density of states, which appears at the bottom of the conduction band. The localised states are separated from itinerant ones by a mobility edge, which is located below Fermi energy. The broad distribution of string sizes can manifest itself as the broad peak of magnetic ac susceptibility around *T* = 55 K^15^,^16^,^17^,^18^.

Thus, a broad variety of the low-temperature “exotic” magnetic phases, observed in magnetic neutron scattering in an external magnetic field^15^ can be associated with the existence of the magnetic clusters as described above. The clusters can merge into string-like droplets or stripy microdomains, and may be ordered or disordered. At low temperatures, the system exists in a glassy state, associated with chaotically distributed ferromagnetic microdomains with the short-range order in the basal plane as seen in the magnetic neutron scattering^15^,^16^,^17^,^18^. At high temperatures, this state survives until the ferromagnetism vanishes in the vicinity of the critical temperature, associated with the self-localisation energy of an electron in the cluster. The similar droplets (fluctuons), originally anticipated in the papers by Krivoglaz^42^,^43^ and for magnetic systems discussed in^44^, can appear as features in the temperature dependence of magnetic susceptibility and resistivity.

## Methods

### Crystal growth

Careful selection and handling of high-purity starting materials and the control of oxygen impurities during the whole preparation process was essential. The polycrystalline feed rods were prepared from bulk pieces of rare earth, transition metal, and silicon of purity 99.9% or better which were arc-melted several times in a water-cooled copper crucible under Ar atmosphere to reach homogenuity. The high-quality single crystals of Tb_2_PdSi_3_ were grown by the floating-zone method with optical heating at melting temperatures > 1500°C from near-stoichiometric polycrystalline feed rods. Post-growth heat treatment was used to improve the actual structure of grown crystals. Composition, microstructure and the perfection of the crystals structure of the samples were investigated by chemical analysis, optical metallography and scanning electron microscopy. The crystallographic orientation of single crystals was determined by the X-ray Laue back scattering method^14^,^45^.

### Experimental approach

In our experiments we used ellipsometry approach. This method offers significant advantages over conventional reflection methods in studying metallic systems as (i) it is self-normalizing and does not require reference measurements, and (ii) full complex dielectric response *ε*(*υ*) = *ω*_1_(*υ*) + i *ε*_2_(*υ*) and the related optical-conductivity spectra, *σ*_1_(*ω*) = *ω ε*_2_(*ω*)/(4*π*), can be obtained directly without a Kramers-Kronig transformation. The complex dielectric response of Tb_2_PdSi_3_ single crystals was investigated in a wide spectral range using a set of home-built ellipsometers at Max-Planck-Institut für Festkörperforschung, Germany. The VIS and UV measurements in the photon energy range of 0.75–6.0 eV were performed with a home-build ellipsometer of rotating-analyzer type, where the angle of incidence is 70.0° For the temperature-dependent measurements, the sample was mounted on the cold finger of a He-flow cryostat with a base pressure of ~ 2 × 10^−9^ Torr at ambient temperature. We used the home-build ellipsometer with incidence angles ranging from 65° to 85° in combination with the *Bruker IFS 66v*/*s* FT-IR spectrometers for the MIR and NIR measurements in the photon energy range of 50 meV −1.0 eV.

## Author Contributions

N.N.K. carried out the measurements and analysed the data. Y.H., W.L. and G.B. synthesised the single crystals. T.N.F. and A.V.B. participated in the data analysis. N.N.K., K.I.K. and F.V.K. wrote the manuscript. K.I.K. and F.V.K. supervised the project.

## Supplementary Material

Supplementary Information Supplementary File
