# Supplementary material for: Formation of metallic magnetic clusters in a Kondo-lattice metal: Evidence from an optical study
Source: Sci Rep. 2012 Nov 27;2:890. doi: 10.1038/srep00890 (PMC3506981; doi:10.1038/srep00890)
Supplement: Supplementary Information — Supplementary File [file srep00890-s1.pdf]

Supplementary online information for the article entitled

Formation of metallic magnetic clusters in a Kondo-lattice metal: Evidence from an optical study

by N. N. Kovaleva, K. I. Kugel, A. V. Bazhenov, T. N. Fursova, W. Löser, Y. Xu, G. Behr & F. V. Kusmartsev

DC transport anomalies

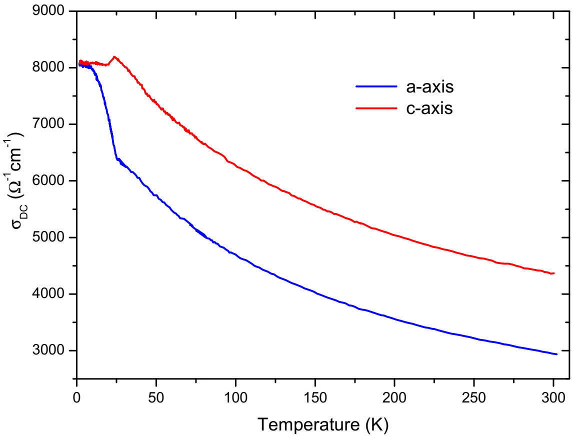

FIG. S1. **DC transport anomalies** Temperature dependencies of dc conductivity in Tb_2PdSi_3 single crystal derived from the transport measurements by Majumdar *et al.* [32].

The dc resistivity measurements in Tb_2PdSi_3 single crystals [32] revealed clear anomalies below the magnetic transition at $T_N = 23.6$ K, as illustrated by the corresponding dc conductivity curves in Fig. S1 . While the **a**- and **c**-polarized dc conductivities follow similar trends, with the **c**-axis conductivity higher by ~ 1700 $(\Omega\cdot\mathrm{cm})^{-1}$ immediately above T_N, the opposite trends appear below T_N, providing evidence that magnetic scattering plays a substantial role in the electronic transport. Surprisingly, the accelerated increase in the **a**-axis conductivity with the simultaneous weak decrease in the **c**-axis conductivity finally results in the isotropic charge transport at low temperatures.

Drude-Lorentz analysis of the dielectric function spectra

To separate the contributions to the complex dielectric response, we performed the classical dispersion analysis by fitting the Drude term and a minimal set of Lorentzian oscillators simultaneously to $\varepsilon_1(\omega)$ and $\varepsilon_2(\omega)$, using a dielectric function of the form [30]

$$\varepsilon(\omega) = \epsilon_\infty - \frac{\omega_p^2}{\omega^2 + i\omega\gamma_D} + \sum_j \frac{S_j\omega_j^2}{\omega_j^2 - \omega^2 - i\omega\gamma_j}.$$

Here ω_p and γ_D are the plasma frequency and the damping of free charge carriers, ω_j, γ_j, and S_j are the peak energy, width, and dimensionless oscillator strength of the jth oscillator, respectively, and ϵ_∞ is the core contribution to the dielectric function. The results of the dispersion analysis of the **ab**-plane and **c**-axis dielectric function spectra are summarized in Table S1.

TABLE I: **Drude-Lorentz analysis.** Parameters of the Drude band and Lorentzian oscillators resulting from the fit to $\varepsilon_1(\omega, T)$ and $\varepsilon_2(\omega, T)$ in the **ab**-plane and **c**-axis. The values of ω_i and γ_i are expressed in eV.

		**ab**-plane		**c**-axis	
	parameters	$T = 10\,\mathrm{K}$	$T = 30\,\mathrm{K}$	$T = 10\,\mathrm{K}$	$T = 30\,\mathrm{K}$
	$\varepsilon_{\mathrm{inf}}$	1.4(1)	1.6(1)	1.9(1)	1.9(1)
Drude	ω_{p}	2.703(5)	2.750(5)	2.464(5)	2.498(5)
	γ_{D}	0.091(2)	0.098(2)	0.096(2)	0.098(2)
mid-infrared	ω_1	0.19(2)	0.19(2)	0.20(5)	0.20(5)
bands	S_{osc}	91(1)	87(1)	151(7)	139(7)
	γ_1	0.26(5)	0.26(5)	0.32(5)	0.33(5)
	ω_2	0.58(2)	0.59(2)	0.57(2)	0.57(2)
	S_{osc}	21.6(2)	22.9(2)	28.1(3)	27.4(3)
	γ_2	0.74(5)	0.76(5)	0.75(5)	0.75(5)
interband	ω_3	1.3(1)	1.3(1)	2.2(1)	2.1(1)
transitions	S_{osc}	2.7(3)	2.9(3)	1.6(2)	1.6(2)
	γ_3	0.6(1)	0.6(1)	1.2(2)	1.2(2)
	ω_4	1.7(1)	1.7(1)	2.7(1)	2.7(1)
	S_{osc}	4.6(5)	4.3(5)	12.8(5)	12.9(5)
	γ_4	0.9(1)	0.9(1)	4.6(3)	4.7(3)
	ω_5	3.4(2)	3.4(2)	5.2(5)	5.2(5)
	S_{osc}	7.9(5)	7.6(5)	0.7(1)	0.6(1)
	γ_5	4.9(3)	4.6(3)	3.1(3)	3.1(3)

Magnetic strings and string glass in Kondo-lattice metals: Theory

Here we show that Kondo-lattice metals have a tendency to form large magnetic string clusters. In a single cluster there are many polarised spins, which can create a potential well that traps several electrons. The magnetic string may be viewed also as a condensation of magnetic polarons into a linear object. The form of the string is arising due to the long-range Coulomb repulsion, which should be taken into account together with the Hubbard on-site repulsion. Such strings may arise not only in the Kondo-lattice metals but also in other systems of narrow band semiconductors. Their creation is inherent to some kind of instability leading to an electronic phase separation associated with local attraction of small magnetic polarons. The magnetic string clusters may also form a tail in the density of states arising at the bottom of the conduction band, which can be detected by spectroscopic methods.

Magnetic polarons in antiferromagnetic semiconductors (ferrons) have been introduced more than 40 years ago [S1]. There, it was assumed that a single charge carrier has been self-trapped by a ferromagnetic or canted antiferromagnetic region. Such magnetic polaron has been originally named as a ferron and had quite a large size. Of course, the size of the ferron may be large or very small. This depends on the kinetic energy of the trapped electron. Large ferrons have a very simple spherical shape [S1-S3]. For smaller ferrons, the structures are more complicated [S4]. The central ferromagnetic region of the ferron consists of a magnetized core, which traps the electron. Its surrounding consists of shells with the oppositely directed moments repelling the trapped electron. The compensating moment of the surrounding oscillates with a period equal to a double lattice constant or larger [S4,S5]. Here, we consider a generalisation of the ferron concept to a multiparticle case, where the central ferromagnetic core traps more than one electron. The formation of such a state stems primarily from the competition between the energies associated with the Coulomb repulsion between electrons and trapping of the particles by the ferromagnetic core. The minimum energy of the Coulomb repulsion is usually associated with a linear shape of the central trapping region of (see, Refs. [S6,S7] for details) and therefore such objects have been named electronic strings [S8,S9] and were detected in oxide materials (see, review article [S10] and references therein). The formation of strings must be enhanced in the ferromagnetic Kondo-lattice metals since the Coulomb interaction there is partially screened. Therefore, in such materials, we expect to observe ferromagnetic electronic strings, which are described below. Indeed, such evidence obtained with the use of the optical method is presented in the main body of the paper.

Here we elucidate that an instability of magnetic polarons may give rise to a creation of a glassy state in the Kondo-lattice metals. That is we have considered small magnetic polarons at zero temperature and found that the small polarons become collapsed into a string cluster consisting of many particles trapped by the spin fluctuation and having the same spin orientation. We investigate this instability taking into account many-body effects of Coulomb interaction for electrons interacting with localised spins ferromagnetically coupled to each other. The long-range

Coulomb interaction is taken into account within the Hartree–Fock approximation. We have found that electrons having the spin orientation parallel with respect to the localized background spins are self-trapped into the droplets. The number of the electrons in a single droplet depends on the ratio of the effective Coulomb and the exchange energies. This state survives until the ferromagnetism vanishes. In the vicinity of the critical temperature the similar droplets (fluctuons) were originally anticipated in the papers by Krivoglaz [S11].

To describe the phenomenon of the droplet or the string formation in the Kondo-lattice metals we start with the Hamiltonian

$$H = t\sum_{ij} c^\dagger_{i\sigma}c_{j\sigma} + \chi\sum_{i\alpha\beta} c^\dagger_{i\alpha}\vec{\sigma}_{\alpha\beta}c_{i\beta}\vec{S}_i + J\sum_{\langle ij\rangle}\vec{S}_i\vec{S}_j - \sum_i \vec{S}_i\vec{h} + H_C, \tag{1}$$

where $\vec{\sigma} = (\sigma_x\sigma_y\sigma_z)$ are Pauli matrices, t is the hopping integral for conduction electrons, which creation and annihilation operators are denoted as $c^\dagger_{i\sigma}$ and $c_{j\sigma}$, respectively. S_i is a Kondo spin localised at the i-th site (for simplicity we consider here a cubic lattice) and h is an external magnetic field. The last term, H_C, is the Hamiltonian of the long-range Coulomb interaction, which is in the Kondo-lattice metals may be screened and replaced by a Hubbard term and a next-neighbour Coulomb repulsion. The constant χ describes an interaction between free electrons and the Kondo spins. The constant J describes a weak exchange antiferromagnetic interaction between Kondo spins. We consider the spins in the classical limit, i.e. we treat the spin operators as classical vectors. This approximation is applicable only for the systems with large values of localized spins, like $S_0 = 3/2, ..., \infty$.

It is convenient to split our discussion into two parts: at first, we will treat only a single particle interacting with localized moments; and then, in the second part we will take into account the Coulomb interaction in the framework of a Hartree–Fock approximation.

The Schrödinger equation describing a single particle interacting with localized magnetic moments has the form

$$-t\Delta\Psi_{\alpha n} + \chi\vec{S}_n\cdot\vec{\sigma}_{\alpha\beta}\Psi_{\beta n} = E\Psi_{\alpha n}. \tag{2}$$

where the Greek subscripts of the wave function $\Psi_{\alpha\beta}$ describe spin indices, like $\alpha = \pm 1$ and $\hat{\Delta}$ is a lattice version of the Laplacian operator, which for the cubic lattice is defined as

$$\hat{\Delta}\Psi_n = -\sum_i(\Psi_n - \Psi_{n+i}), \tag{3}$$

where the summation is carried out over all the nearest-neighbor sites around the n-th site; here, for a simplicity, the spin index α is omitted. With the use of this equation one can construct the total energy of the system, which includes the electronic energy E and the exchange energy corresponding the ferromagnetically localised spins, described by Heisenberg Hamiltonian H_H, i.e.

$$F = E(S_1, ..., S_N) + J\sum_{<ij>}\vec{S}_i\vec{S}_j - \sum_i\vec{S}_i\vec{h}, \tag{4}$$

where we choose $J > 0$. As a reference state, we consider a ferromagnetic state formed by localised spins $\vec{S}_i = S_0\vec{l}$, with $\vec{l}\cdot\vec{l} = 1$. Now let us consider self-trapping of the electrons by a magnetic fluctuation. If the coupling constant χ/J is large, the electron can significantly disturb the spin background. The maximum value corresponds to the spin flip. Therefore, the spin configuration of a fluctuation at the self-trapping will take the form

$$\vec{S}_{n0} = \begin{cases} \quad -S_0\vec{l}, & \text{if} \quad 1 \le \mathrm{n_x} \le \mathrm{N} \\ \equiv \quad S_0\vec{l}, & \qquad \text{otherwise.} \end{cases} \tag{5}$$

Note that at large value χ/J this fluctuation has a sharp boundary.

After substituting this solution into the Schrödinger equation, we get the following system of equations

$$-t\Delta\Psi_{\alpha n} + \chi\vec{\sigma}_{\alpha\beta}\cdot\vec{S}_{n0}\Psi_{\beta n} = E\Psi_{\alpha n}. \tag{6}$$

It is convenient to choose the system of coordinates with z axis oriented along the magnetic moments of the ferromagnets, i.e. along $\vec{l}$. In this system of coordinates, these equations for the electrons trapped by the magnetic fluctuation are simplified to

$$\begin{cases} -t\Delta\varphi_n = (E + \chi S_0)\varphi_n & \text{if} \quad 1 \le \mathrm{n_x} \le \mathrm{N} \\ -t\Delta\varphi_n = (E - \chi S_0)\varphi_n, & \qquad \text{otherwise} \end{cases} \tag{7}$$

and analogous equation for electrons with other polarisation f_n, i.e. the electron wave function was taken as $\vec{\Psi_n} = (\varphi_n, f_n)$. These equations describe a particle localised in a square and flat potential well of the deepness $-\chi S_0$ and the height $+\chi S_0$. If we assume that this potential well is infinitely deep and trapped electrons are spin polarised, the solution takes the simple form; the wave function does not vanish if the index n corresponds to sites within the fluctuation, otherwise it vanishes, i.e. in the limit $\chi/J \to \infty$ the system of equations (7) has the following exact solutions: $f_n \equiv 0$ and

$$\varphi_{nx}(k_x) = \begin{cases} \frac{1}{\sqrt{N}} \exp(ik_x n_x), & \text{if} \quad 1 \leq \mathrm{n_x} \leq \mathrm{N} \\ \equiv 0, & \text{otherwise.} \end{cases} \tag{8}$$

This single particle wave function corresponds to the following eigenvalue

$$E = -\chi S_0 + Zt - 2t\cos(k_x), \tag{9}$$

where Z is a number of nearest neighbours. Note that the large term Zt associated with the confinement of the electrons may be compensated by a coupling with the ferromagnetic spins of the string $-\chi S_0$.

This solution has a cigar (or string) shape. Such form of the localised state is dictated by the Coulomb interaction, which has the smallest value for the linear string shape [S6,S7]. The described magnetic string is a generalization of a magnetic "polaron", which was originally anticipated in the papers by Krivoglaz [S11] and Nagaev [S12]. Krivoglaz has called "fluctuon" an analogous state of a spherical shape created in the vicinity of the critical point of a ferromagnetic phase transition [S11] by a cigar shape fluctuation consisting of the flipped magnetic moments is minimal.

Thus, an important role in the formation of the many-particle state plays the Coulomb interaction which is trying to remove the trapped particles from the trapping well associated with the magnetic fluctuation. Of course, if M particles are trapped by the potential well, they occupy the energy levels of the well according to the Pauli principle, i.e. on the each energy level will be only one particle. On the other hand, in this case, the potential well of such a fluctuation will be created by all M particles and therefore will be M times deeper and wider than in the case of the single particle state.

Thus, in this many particle self-trapping each of M particles having the up-spin is localised in the potential well self-consistently created by all trapped particles and each particle occupies only a single energy level inside the well. Having these single particles wave functions (Eq. (8)) we are now in a position to take into account the long-range Coulomb interaction to apply the Hartree–Fock approximation. With the use of these single particle wave functions we built up the many-body Hartree–Fock wave function. The many-body wave function of the M particles $\Psi(1, 2, ..., M)$ self-trapped in the string and whose spins are polarised is taken to be in the form of a Slater determinant of single particle wave functions, Eq. (8)

$$\Psi(1, 2, ..., M) = \frac{1}{\sqrt{M!}} \det \mid\mid \psi_i(k_j) \mid\mid . \tag{10}$$

Such a kind of wave functions is proved to be good to describe the Coulomb correlations in the Hubbard and $t - J$ models [S13].

Then the Coulomb contribution into the total energy of M trapped particles is estimated as

$$E_c = \langle \Psi \mid \sum_{i<j} \frac{1}{(r_i - r_j)} \mid \Psi \rangle. \tag{11}$$

To perform such an averaging with the use of the Hartree–Fock many-body wave function, first, we have calculated the pair correlation function. With the aid of the pair correlation function we have calculated the dependence of the Coulomb energy on N and M given by

$$V_{HF} = V \frac{4M^2}{N} \int_0^{\pi} \frac{dx}{x} \left(1 - \frac{\sin^2(Mx)}{M^2 \sin^2 x} \right), \tag{12}$$

where $V = \frac{e^2}{2\bar{\epsilon} a}$ and the parameter $\bar{\epsilon}$ is the effective dielectric constant. The numerical estimation of the integral in Eq. (12) shows that a function V_{HF} behaves similarly to that obtained independently in the electrostatic approximation, where we assume that the M point charges are equidistantly located in the string. For such an assumption the energy contribution from the long-range part of the Coulomb interaction is straightforward to calculate. The long-range part of the Coulomb interaction of M particles with the charge e, separated by a distance aN/M and self-trapped into a string of length N is approximately equal to

$$E_C \approx V \left(\frac{M^2 \log M}{N} \right). \tag{13}$$

The momentum $\mathbf{k}$ of electrons with polarised spins is simply quantised if we assume that their wave function satisfies periodic boundary conditions (PBC) along the string. If we employ other boundary conditions for the trapped electrons (for example, open boundary conditions), the main result will not change drastically.

With the use of PBC the electron momenta along the string are quantised, $k_{nx} = 2\pi n/(aN)$. With the use of Eq. (9) and the Pauli exclusion principle, we calculate the expression for the adiabatic potential $J_{N,M}$ describing M trapped electrons

$$J_{N,M} = ZtM - \chi S_0 M - 2t\frac{(N-1)\sin(\pi M/N)}{N\sin(\pi/N)}. \tag{14}$$

If the last two terms in Eq. (14) are larger than the first one, with the increase of the number of trapped particles, M, the value of the electron energy or the adiabatic potential $J_{N,M}$ decreases. This suggests the possibility of the electronic phase separation in the Kondo-lattice metal and the formation of a large linear object. However, the size of these objects is limited by the Coulomb interaction H_C (see, the expression for the total Hamiltonian, Eq.(1)).

For the next illustration, to estimate the total string energy, F_S, we use E_C instead of V_{HF} and add this term to the expression for the adiabatic potential (14). Because of the negative contribution to V_{HF} arising from the exchange forces, we gave $V_{HF} \leq E_C$. Such a substitution is, therefore, justified if we are interested in estimating lower limit for the string size due to the Coulomb energy. Also it gives the explicit expression Eq. (13), which is convenient for the analysis.

As a result, the total energy F_S, which consists of the electron energy $J_{N,M}$ and the energy of the Coulomb repulsion E_C, equals

$$F_S = -\chi S_0 M + ZtM - 2t\frac{(N-1)\sin(\pi M/N)}{N\sin(\pi/N)} + V\left(\frac{M^2\log M}{N}\right). \tag{15}$$

Here, in the simplest approximation, we neglected the magnetic energy associated with the Heisenberg Hamiltonian H_H, related to the last two terms in our Hamiltonian (see Eq. (1)). The presented expression for F_S (Eq. (15)) has an absolute minimum at some fixed number of particles M self-trapped into a string bag having an optimum length, N. In insulators, the equations, which determine the optimum values of M and N are obtained by minimization of F_S with respect to M and N. In metals, the value of F_S should be compared to the Fermi energy, E_F, that is for each value of N, the value of M should be determined from the equation $F_S(N,M) = E_F$. In the case of the insulating ferromagnetic string, when $N = M$, we can immediately obtain the equation to determine the number of the trapped electrons, M

$$-\chi S_0 + Zt + \log M = E_F, \tag{16}$$

whence we find

$$M = \exp\left(\frac{E_F + \chi S_0 - Zt}{V}\right). \tag{17}$$

Note that in the Kondo-lattice metals with large Fermi surface the magnetic strings may have a very large length since the characteristic potential of the Coulomb interaction will be screened on large distances and may be very small. Typically, it is described by the the Coulomb potential associated with the next-neighbour interaction.

In general, the strings with the energy lower than the Fermi energy will be also created, and form the Lifshiz tail in the density of states which may be spread well in the bandgap.

The comparison of the string energy F_S per electron (see Eq. (15)) with the Fermi energy indicates that a string of the length N with M trapped particles may have a total energy (including electronic, magnetic, and Coulomb contributions), which is lower than the Fermi energy or the total energy of M separated magnetic polarons [S1]. Therefore, the state associated with M magnetic polarons (either separated, forming a Wigner crystal, or forming a Fermi liquid) is unstable and will collapse into a mixture of different strings forming a complicated magnetic order.

The described linear strings will also have much lower energy than sheet configurations, like a single circular or square spot (droplet), consisting of M sites inside the droplet with M self-trapped particles. It is clear, for example, from the simple electrostatic arguments presented that for the configuration of a single circular droplet a contribution to the total energy from the Coulomb interaction is strongly increased compared to a string configuration, while a contribution from the exchange interaction being short-ranged remains the same. Although these droplets have higher total energy than strings per particle, it is still smaller than the energy of a single small polaron.

Thus, we arrive at the conclusion that in Kondo-lattice metals **small magnetic polarons are unstable; this instability induces the formation of strings, which may be ordered or form highly disordered glassy state**. The similar state was discussed earlier for the case of pyrochlore manganites [S14]. These strings are created by an exchange interaction between free particles and localised Kondo magnetic moments. The string may not only have a linear form but may be also bent, curved or even have a shape of a closed loop. Such curved configurations will probably correspond to low-energy excitations of the string.

The concept of the string arising in Kondo-lattice metals introduced here is very general. Depending on the fermion filling of the strings, the individual strings may be both insulator and metallic, which are coexisting with each other. Such a large variety of the different types of strings may give rise to different novel effects which could arise in materials with narrow bands: both semiconductors and metals. In general, for any narrow band Kondo-lattice metal, we expect the formation of a new type of the electronic order formed by the long linear objects – strings. There, the criterion for the string formation is significantly improved, because in the Kondo-lattice metals the total energy of the strings is comparable or lower than the Fermi energy, from one side, and the Coulomb repulsion is significantly screened, from the other side. It seems that for such metals it is a common phenomenon that there is a coexistence of strings and free fermions, which balance is dictated by an interplay of Fermi energy, Coulomb and exchange forces. These complicated issues will be discussed in our future publications.

In summary, we demonstrate here that Kondo-lattice metals represent an example of a new electronic state of matter where there is a coexistence of the magnetic clusters with free fermions. The magnetic moments associated with individual clusters may be disordered at high temperatures and ordered in a some kind of antiferromagnetic order at low temperatures. The antiferromagnetic-type order is associated with dipole character of magnetic interaction between the total magnetic moments of individual clusters. In general, we expect that the low-temperature state has a glassy character. The application of an external field increases the length of the strings and therefore increases a size of ferromagnetic domains, hence, diminishing the spin fluctuation scattering in the sample.

Acknowledgements. We are very grateful to E. I. Rashba for illuminating discussions.

Supplementary references

[S1] Nagaev, E. L. *Pisma v Zh. Eksp. Teor. Fiz.* **6,** 484 (1967). [*JETP Lett.* **6,** 18 (1967)]; *Zh. Eksp. Teor. Fiz.* **54,** 228 (1968). [*Sov. Phys. JETP* **27,** 122 (1968).]
[S2] Mott, N. and Zinamon, Z. *Rep. Prog. Phys.* **33,** 81 (1970).
[S3] Nagaev, E. L. *Phys. Reports* **346,** 388 (2001).
[S4] Nagaev, E. L. *Pisma v Zh. Eksp. Teor. Fiz.* **74,** 472 (2001). [*JETP Lett.* **74**, 431 (2001).]
[S5] Nagaev, E. L. *Phys. Rev. B* **66,** 104431 (2002).
[S6] Kusmartsev, F. V. *J. de Physique IV* **9,** Pr10-321 (1999).
[S7] Kusmartsev, F. V. *Phys. Rev. Lett.* **84,** 530 (2000); *Phys. Rev. Lett.* **84,** 5026 (2000).
[S8] Kusmartsev, F. V., Di Castro, D., Bianconi, G. & Bianconi, A. *Phys. Lett. A* **275,** 118 (2000).
[S9] Kusmartsev, F. V. *Int. J. Mod. Phys. B* **14,** 3530 (2000).
[S10] Kusmartsev, F. V. *Contemp. Phys.* **45,** 237 (2004).
[S11] Krivoglaz, M. A. *Usp. Fiz. Nauk* **113,** 617 (1973). [*Sov. Phys. Uspekhi* **16,** 856 (1974).]
[S12] Nagaev, E. L. *Physics of Magnetic Semiconductors* (Mir Publishers, Moscow, 1983).
[S13] Kusmartsev, F. V. *Phys. Rev. B* **43,** 6132 (1991).
[S14] Kusmartsev, F. V. *Physica B* **284-288,** 1422 (2000).
